# Comparison of Polypropylene Sling with Combined Transconjunctival Retractor Plication and Lateral Tarsal Strip for Correction of Involutional Lower Eye Lid Ectropion

**DOI:** 10.2174/1874364101711010285

**Published:** 2017-08-31

**Authors:** Ruchi Goel, Abhilasha Sanoria, Sushil Kumar, Deepanjali Arya, Smriti Nagpal, Neha Rathie

**Affiliations:** Department of Ophthalmology, Gurunanak eye center, Maulana Azad Medical College, New Delhi-110002, India

**Keywords:** Involutional, Ectropion, Transconjunctival retractor plication, Lateral tarsal strip, polypropylene sling, Epiphora

## Abstract

**Purpose:**

The study aims to compare the effectiveness and complications of transconjunctival retractor plication (TRP) with lateral tarsal strip (LTS) and the polypropylene sling (PS) surgery for treatment of involutional lower lid ectropion.

**Method:**

A prospective randomised pilot study was conducted on 30 eyes of 30 patients suffering from epiphora having horizontal eyelid laxity >6mm and age >50 years at a tertiary care centre from December 2014 to March 2015. They were randomly divided into two equal groups for TRP with LTS (group A) and PS (group B). Success was defined as relief in epiphora and lid laxity ≤4mm at 12 months post operatively.

**Result:**

There were 19 male and 11 female patients with age ranging from 55-80 years. The mean grade of ectropion was 2.80±1.32 in group A and 2.87±1.60 in group B. The preoperative horizontal laxity increased with the grade of ectropion (p <0.001) while medial canthal laxity was variable. The average surgical time per procedure in group A was 66 minutes and in group B was 24 minutes. Group A had a success rate of 93.33%, while group B had a success rate of 87%. Post-operative complications occurred in 2 eyes in group B only.

**Conclusion:**

Both LTS with TRP and PS are effective in the management of involutional ectropion. LTS with TRP though more invasive has higher success rates and a lower incidence of complications as compared to PS. However, PS is an easy to perform out- patient procedure that is faster and better tolerated in old patients.

## 1 INTRODUCTION

Lower eyelid involutional ectropion is a common cause of epiphora in elderly population. Occurrence of senile ectropion involves interplay of various factors namely laxity of the suspensory canthal tendons, ischaemia and atrophy of the pretarsal and preseptal orbicularis muscle which result in laxity of lower lid structures [1]. The horizontal lid laxity can be generalized or may involve medial or lateral canthus. As the ectropion progresses, exposure causes secondary inflammatory changes in the conjunctiva and thickening of tarsus, that further worsen the ectropion. Marked ectropion may result in lagophthalmos with resultant corneal exposure and in extreme cases, corneal ulceration thereby justifying an early intervention [2].

Management of involutional ectropion is directed towards shortening the lid in the area of maximal laxity to bring it in apposition with the globe. If the laxity is generalized, a pentagonal wedge resection is performed, combined with blepharoplasty if required. If the lid laxity is more lateral, it is addressed by Lateral Tarsal Strip (LTS) or full thickness lid shortening performed laterally. With medial lid laxity full thickness lid resection, medial canthal suture or tarsoconjunctival diamond excision is employed in the medial part of the lid [1, 3]. An alternative minimally invasive technique using polypropylene suture that is Polypropylene sling (PS), whereby a horizontal non-absorbable suture is passed in the pre-tarsal plane from lateral canthal ligament to medial canthus to tighten the lid, has been used successfully for the treatment of epiphora in mild and moderate involutional ectropion [4].

The classical procedures involving the excision of the tarsal plate are associated with many complications like shortening of horizontal palpebral fissure, eyelid notching and lower eyelid retraction. Thus to avoid these complications, transconjunctival retractor plication with tarsal strip (TRP with LTS) is gaining popularity due to its high success rates [5]. On the other hand, PS is a minimally invasive procedure which makes it suitable for old population suffering from multiple co-morbidities. This study will compare the effectiveness and complications of two surgical approaches *i.e.* TRP with LTS and the PS procedure for the treatment of involutional lower lid ectropion.

## 2 METHODS

After receiving approval from institutional ethical committee and informed consent of each patient, a prospective randomised pilot study was conducted on 30 eyes of 30 patients suffering from epiphora due to involutional lower lid ectropion, having horizontal eyelid laxity > 6mm and age > 50 years attending the outpatient department and oculoplasty clinic of a tertiary care centre from December 2014 to March 2015. Patients with blocked lacrimal passages, previous lid surgery, severe dry eye, trichiasis, blepharitis, skin scarring, uncontrolled systemic illness and those not willing for follow up, were excluded from the study.

The study population was randomly distributed into two equal groups by computer generated tables and group 1 underwent TRP with LTS and group 2 PS surgery. All the patients underwent complete work up. A detailed history was taken regarding the nature of watering, lid trauma, facial palsy, lid swellings to rule out other types of acquired ectropion, previous lid surgery or any systemic illness. Assessment of best corrected visual acuity, examination of ocular surface, conjunctiva, cornea, anterior chamber, to rule out causes of hyperlacrimation and lid examination were done to look for scar mark suggestive of previous trauma or surgery.

The eyelid laxity was assessed as follows: (Figs. **1a**-**c**)

1. Horizontal laxity by pinch test
2. Lateral distraction test for medial canthal tendon laxity
3. Lateral canthal tendon laxity
4. Inferior lid retractor laxity
5. Position of puncta on up gaze and primary gaze

The amount of ectropion was graded according to the ectropion grading scale proposed by Moe and Linder as follows [6]

0- Normal eyelid appearance and function
1- Normal appearance but symptomatic; eyelid laxity present on examination
2- Scleral show without eversion of lower eyelid
3- Ectropion without eversion of lacrimal punctum from lacrimal lake.
4- Advanced ectropion with eversion of lacrimal punctum from lacrimal lake.
5- Ectropion with complications (*e.g.* conjunctival metaplasia, retraction of anterior lamella or stenosis of lacrimal system).

Blood investigations such as haemogram, bleeding time, clotting time and random blood sugar were performed. All surgeries were performed under local anaesthesia by a single surgeon (first author).

Transconjunctival retractor plication and lateral tarsal strip (Figs. **2a** to **d**).

- Aseptic preparation and draping of the eyes were carried out and a local infiltration of 2% lignocaine with 1:100000 adrenaline was administered into the lower eyelid and along the lateral orbital wall
- Traction sutures were applied in the lower lid
- A horizontal incision was made along the lower border of the tarsal plate transconjunctivally
- This incision was extended 2 mm medial to the punctum and medial to the midpoint of the eyelid laterally
- The retractors were identified by their movement when the globe moved up and down and then reattached to the lower edge of tarsus with 6-0 polyglactin suture (Vicryl, Ethicon Inc)
- This was followed by Lateral Tarsal Strip procedure where the inferior limb of the lateral canthal tendon was first crushed and then incised
- A new tendon was fashioned by excising the skin, orbicularis, lashes and conjunctiva from the tarsus as far as the proposed position of the new lateral canthus
- This tarsal strip was then attached to the periosteum at the level of lateral tubercle with a double-armed 5-0 polypropylene suture

- The canthus was reformed, and the wound closed with 5-0 polyglactin suture (Vicryl, Ethicon Inc) and 6-0 silk suture Polypropylene sling (Figs. **2e**, **f**).

- Aseptic preparation and draping of the eyes were carried out and a local infiltration of 2% lignocaine with 1:100000 adrenaline was administered into the lower eyelid, medial canthal angle and along the lateral canthal angle
- A horizontal incision of 2 mm was made over the lateral orbital wall. The orbicularis muscle was dissected to expose the lateral canthal tendon
- A 5-0 polypropylene suture (Prolene, Ethicon Inc) was tied to the attachment of lateral canthal tendon and adjoining periosteum at lateral orbital rim
- A 3mm vertical incision was then placed medial to the medial canthus and the anterior and posterior limb of medial canthal tendon were exposed
- A spatula was inserted between the lid and the globe
- The other end of the 5:0 polypropylene suture was mounted on a long straight needle and passed through the length of the lid in the pretarsal plane, keeping it as close to the lid margin as possible
- The needle was then taken out at the medial incision behind the anterior limb of medial canthal tendon
- Traction was applied on the suture to cause, tightening of the lid margin to the globe
- The straight needle was replaced with a semicircular one and the 5:0 polypropylene suture was tied to the attachment of posterior limb of medial canthal tendon and adjoining periosteum at posterior lachrymal crest. The suture was pulled till there was dipping of the inferior punctum into the lacrimal lake
- The orbicularis muscle was closed with 6-0 polyglactin suture (Vicryl, Ethicon Inc) and skin incisions were closed with 6-0 silk suture

**Post Op Treatment**

Tablet Ciprofloxacin (500 mg 12 hourly), Tablet Ibuprofen (400mg 8hourly for 5 days, Topical antibiotics (Ofloxacin 0.3%) 6 hourly for 2 weeks and Tablet Serratiopeptidase 10 mg TDS for 3 days. Silk sutures were removed on the 10th postoperative day.

The patients were followed up on day 1, day 10, every month for 6 months and then at 12 months. Complications like lid edema, lid contour abnormality, exposure keratitis, suture infection, *etc* were noted. Assessment of under correction/overcorrection was done and clinical photographs were taken.

### 2.1 Outcome Variable

- Primary outcome: Success was defined as relief in epiphora and lid laxity ≤4mm at 12 months post operatively.
- Secondary outcome: Occurrence of undercorrection, overcorrection and any complications

### 2.2 Statistical Analysis

- The success rate and other qualitative variables in both the groups was expressed in terms of frequencies and percentages and compared using chi-square/fisher’s exact test. The p-value of <0.05 was considered statistically significant. SPSS (Statistical package for social sciences) version 15-0 software was used for the analysis.

## 3 RESULTS

Thirty eyes of thirty patients suffering from lower lid involutional ectropion were included in the study of which 15 underwent TRP with LTS (group A) and in 15 PS (group B) was performed. The age ranged from 55-80 years with a mean age of 69.33±6.41 years in the group A and 70.53±6.09 years in group B. Majority of patients belonged to 60-70 years of age. The two groups are age matched (p = 0.302 using independent t-test).

Out of the 30 patients, there were 19 male and 11 female patients comprising 63.33% and 36.66% respectively. The two groups were sex matched (p=0.352). In 18 (60.0%) patients right eye was operated and in 12 (40.0%) patients left eye was operated.

The mean grade of ectropion was 2.80±1.32 in group A and 2.87±1.60 in group B (p = 0.451) (Table **1**).

The preoperative horizontal laxity in patients was found to progressively increase with the grade of ectropion (p <0.001 using the chi-square test) while medial canthal laxity was variable. Just one patient in the study had lateral canthal laxity and had grade 4 ectropion. Inferior lid retractor laxity was present in 5 eyes; 4 eyes (26.67%) had inferior lid retractor laxity in Group A and 1 eye (6.67%) had inferior lid retractor laxity in Group B. Mild ectropion with punctal apposition but symptomatic epiphora was present in 16 cases, of which, 66.67% were in Group A and 40% cases in Group B. There was no significant difficulty in surgical procedure in either of the two groups. There were no major intra-operative complications.

The surgical time in group A was approximately 66 minutes per procedure (1.14 hours) much greater than group B that was approximately 24 minutes (0.38 hours) also, Polypropylene sling was found to be an easier procedure to perform and could be done on an out-patient basis.

At one year of follow up, Group A had a success rate of 93.33%(14 out of 15 eyes), while group B had a success rate of 87%(13 out of 15 eyes). (Table **2**) All patients in both the groups had their puncta apposed to the globe at 12 months but in failed eyes horizontal lid laxity was greater than 4mm. Figs. (**3a**, **d**). There was no difference in the success rate between the two groups statistically (p = 0.271 fisher exact test).

No complications were seen in Group A patients whereas, 2 eyes in group B had complications postoperatively. Out of these two patients one developed lateral ectropion on post op day one that persisted till day 10 and was followed by reversion to the original pre-operative laxity which was seen on follow up at 4th week. Figs. (**4a**, **b**). This occurred due suture slippage that led to the reappearance of the preoperative laxity measurement. The patient refused resurgery following which no intervention was done. In the other patient lateral entropion grade 1 (with inner margin inturned) developed at 5th month. Fig. (**5a**, **b**) As, the patient was not symptomatic no intervention was done. Suture erosion was not seen in any of the cases. The relation between the occurrence of complications and the grade of lid laxity was not statistically significant (p = 0.928 chi-square test). The comparison of occurrence of complications in the two groups was not statistically significant (p = 0.072).

## 4 DISCUSSION

Involutional lower lid ectropion results in troublesome epiphora in elderly. Majority of patients belonged to 60-70 year age group in our study. Ghafouri *et al.* had earlier studied the effect of TRP with LTS with average age in their study population being 82.2±5.9 years [2].

Preoperatively, the horizontal laxity was found to progressively increase with the grade of ectropion while medial canthal laxity was variable. Just one patient in the study with grade 4 ectropion had lateral canthal laxity. The relation between the horizontal lid laxity and grade of ectropion was statistically significant (p <0.001chi-square test).

Intraoperatively, there was no significant difficulty in surgical manipulation in either of the two procedures. However, the average surgical time of LTS with TRP was found to be much longer (approximately 66 minutes per procedure in comparison to 24 minutes in Polypropylene sling). Also, Polypropylene sling was easier to perform and could be done on an OPD basis in comparison to LTS with TRP that was relatively more invasive involving extensive tissue dissection and utilizing a greater amount of surgical skill.

The lower eyelid helps to circulate the tears from its origin in the lacrimal gland to its drainage into the larimal puncta [7]. Tear drainage through the lacrimal excretory system involves both active and passive mechanisms [8]. The lacrimal pump actively sucks tears into the lacrimal sac with each blink. The contraction of the orbicularis pulls the lower punctum medially, closes the ampulla, and displaces the lateral wall of the lacrimal sac laterally, creating negative pressure in the sac that draws tears from the common canaliculus into the sac. The pump's effectiveness decreases with age, probably, as a result of decreasing orbicularis tone, increasing fibrosis in the nasolacrimal drainage system and alterations in the canthal tendon anatomy. By tightening the eyelid, the orbicularis muscle functions more effectively to close the eyelid and propel tears towards the punctum. Eyelid tightening is also proposed to increase the pull on the walls of the lacrimal sac, creating a pressure difference to aid the entry of tears into the sac [9]. Thus, lid tightening should be performed even if the punctum is apposed in symptomatic patients. This was documented in our series where epiphora was relieved in grade 1 ectropion.

Various procedures have been tried for the correction of involutional ectropion depending on the amount and location of laxity. Since the etiology involves multiple factors, no ideal single technique has been agreed upon. LTS procedure can be done alone or in combination with TRP or medial spindle, with variable results. TRP and medial spindle invert the lower punctum by strengthening the inferior lid retractors [10]. TRP with LTS has the advantage of achieving horizontal tightening and addressing the dehiscence of lower lid retractors without excision of posterior lamellar tissue. It has been shown to be 100% effective in patients with medial ectropion [5].

Rocca [11] has reported various complications of the LTS procedure like overriding of the upper eyelid, blunting of the lateral canthal angle, creation of gap between the eyelid and the globe by too anterior placement, occurrence of problems of ocular exposure and watering by too inferior placement of the tarsal strip. None of these complications were seen in our study due to appropriate positioning by an experienced surgeon.

Since LTS alone was associated with the problem of displacement of puncta in cases of medial canthal laxity, its combination with TRP has a more favourable outcome. This could be explained by the fact that in LTS, the forces pull the lid margin laterally and backwards, there being no medial stabilization. TRP, on the other hand pulls the lower border of the tarsus outwards, facilitating inward movement of the lower punctum.

PS though does not address the vertical imbalance but, the horizontal forces act both medially and laterally. The medial tightening directs the forces postero-medially, thereby correcting the direction of the punctum which is mainly important for tear drainage and relief from watering.

In our study, success in terms of apposition of puncta, correction of horizontal lid laxity <4mm and relief of symptoms was attained in the LTS with TRP group in 93.33% of patients while, success was achieved in 87% of patients who underwent the Polypropylene sling procedure.

No complications were seen in the LTS with TRP group patients whereas, 2 patients of Polypropylene sling group had complications postoperatively. The relation between the occurrence of complications and the grade of lid laxity was not statistically significant (p value -0.928 calculated using the chi-square test). Out of the two patients in group B, one patient developed lateral ectropion on the immediate post-operative day that could be attributed to the lower level of suture placement intraoperatively. Subsequent cheese wiring of the suture occurred that led to the reappearance of the preoperative laxity measurement. In the second patient, lateral entropion developed at 5th month of follow up which persisted till the last visit. The entropion had the inner lid margin inturned (grade1) and as the patient was asymptomatic, no intervention was done. Thus, the level of suture placement is of great importance in this procedure as placement at a lower level could result in ectropion and too high suture placement could lead to an entropion as noted in this study. The suture should be placed about 2mm from the lid margin and correct positioning ensured intraoperatively. None of the patients developed suture exposure or suture granuloma as reported previously [4]. This was achieved by meticulous closure of the orbicularis muscle and skin separately over the suture knot.

Though we did not get a statistically significant difference between the two groups in terms of success rates and complications, it could be due to a small sample size in our study. Thus, this study showed that LTS with TRP though more invasive, but still stands to be a better procedure with better success rates and a lower incidence of complications as compared to PS. However, PS scored over LTS with TRP, being an easy to perform procedure on out- patient basis that requires much lesser time and is better tolerated in old patients. PS can thus be especially useful in old patients with co morbidities where shorter surgical time is preferable.

## Figures and Tables

**Fig. (1a):**
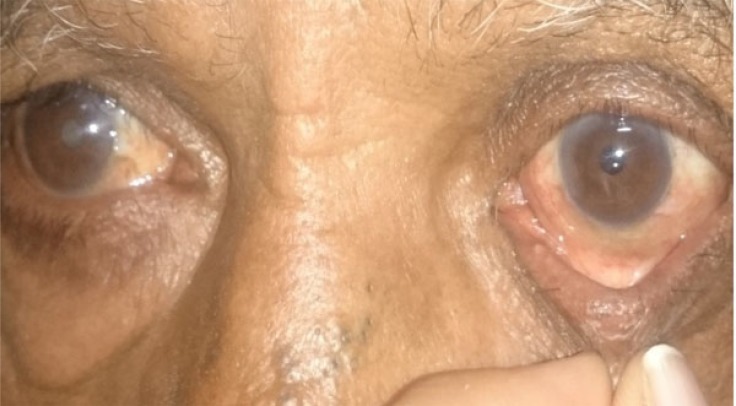
The lower lid is distracted away from the globe. A distance of more than 6mm or more is considered abnormal and confirms horizontal laxity.

**Fig. (1b):**
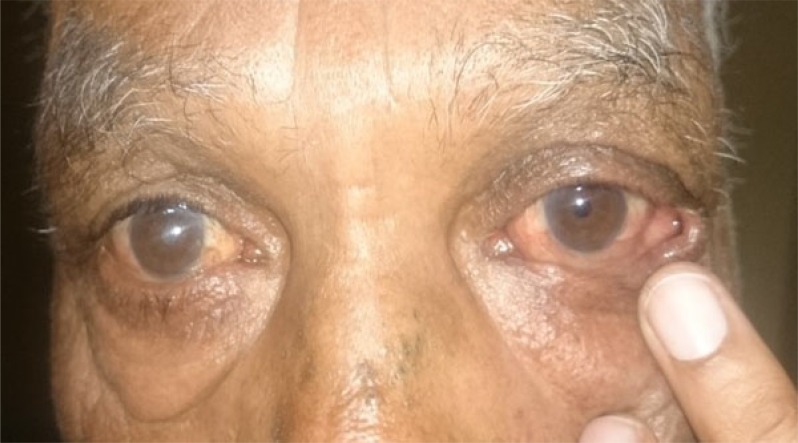
Lateral distraction test showing medial canthal laxity.

**Fig. (1c):**
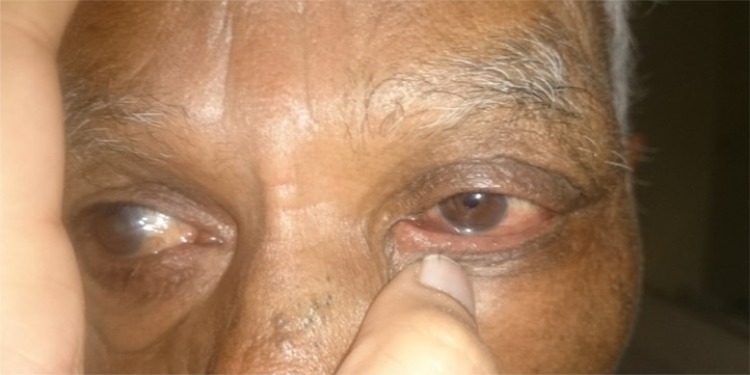
Test for lateral canthal lid laxity.

**Fig. (2a):**
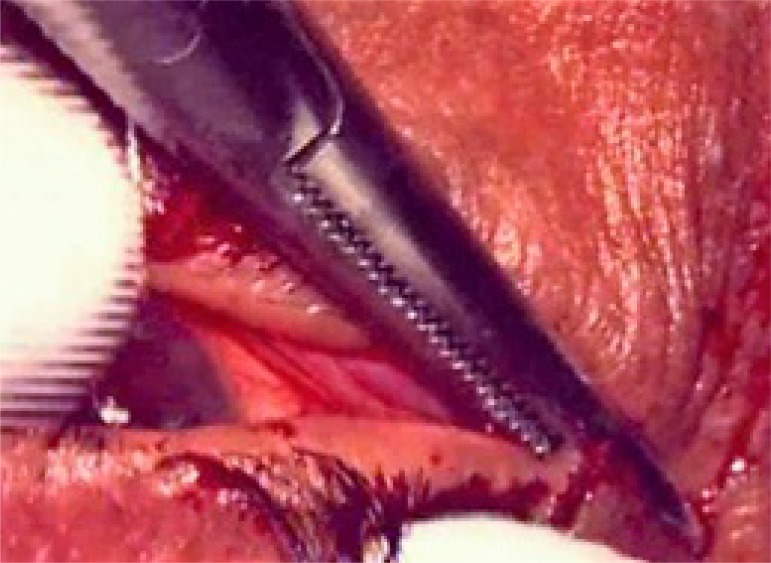
TRP with LTS :Lateral canthotomy being performed.

**Fig. (2b):**
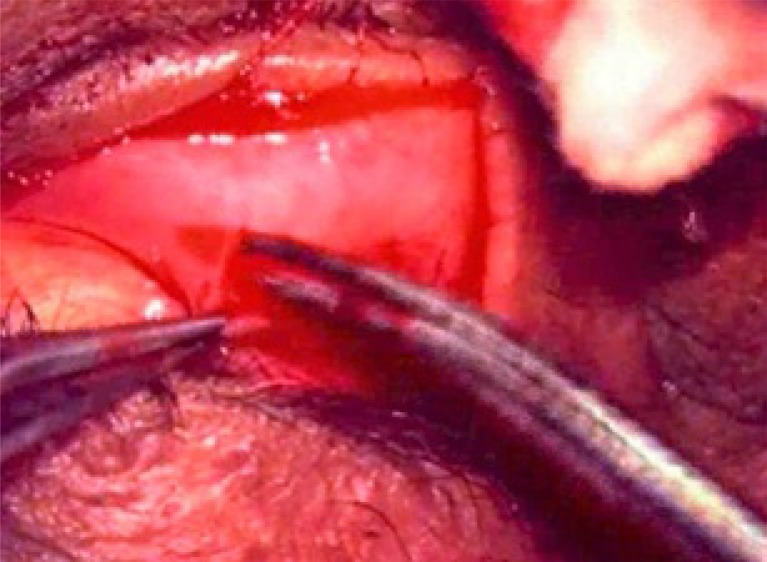
TRP with LTS : Horizontal incision was made along the lower border of the tarsal plate transconjunctivally.

**Fig. (2c):**
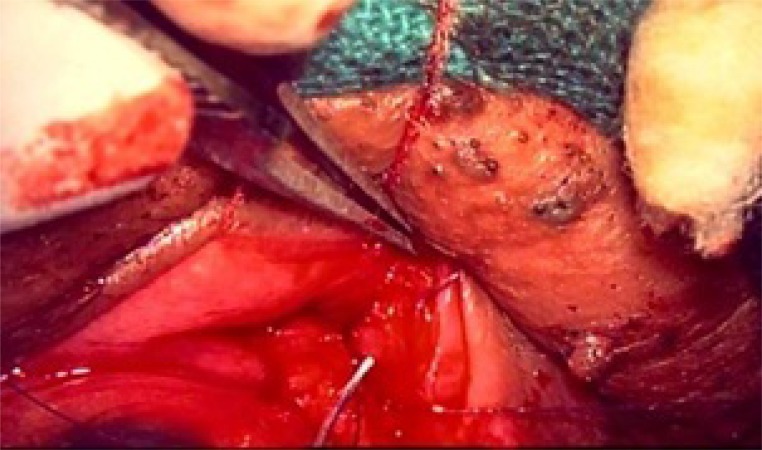
TRP with LTS: Retractors being reattached to the lower edge of tarsus.

**Fig. (2d):**
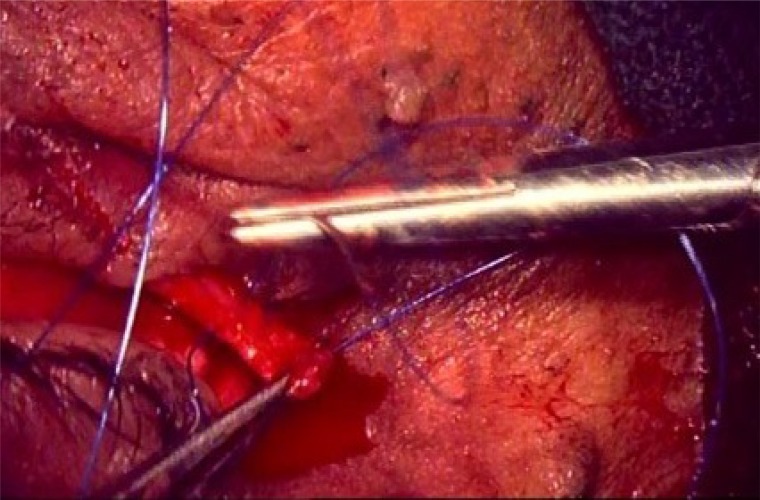
TRP with LTS: Prolene suture being passed through the fashioned strip of tarsus for anchorage to periosteum.

**Fig. (2e):**
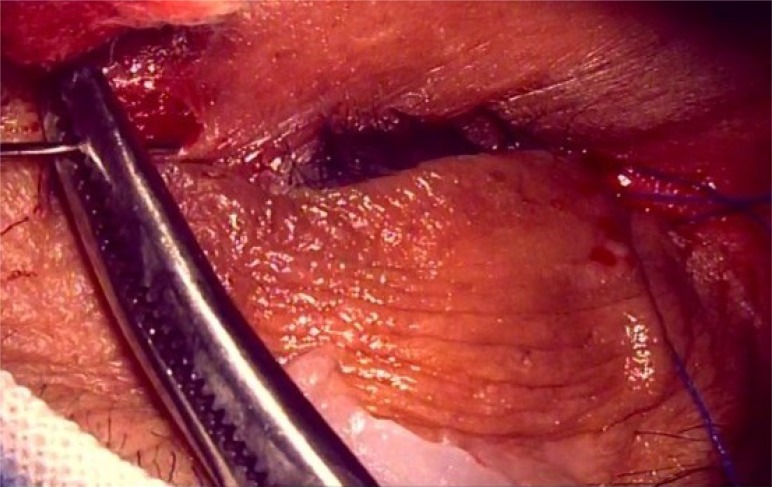
PS:A 5-0 polypropylene suture (Prolene, Ethicon Inc) was tied to the attachment of lateral canthal tendon and adjoining periosteum at lateral orbital rim.

**Fig. (2f):**
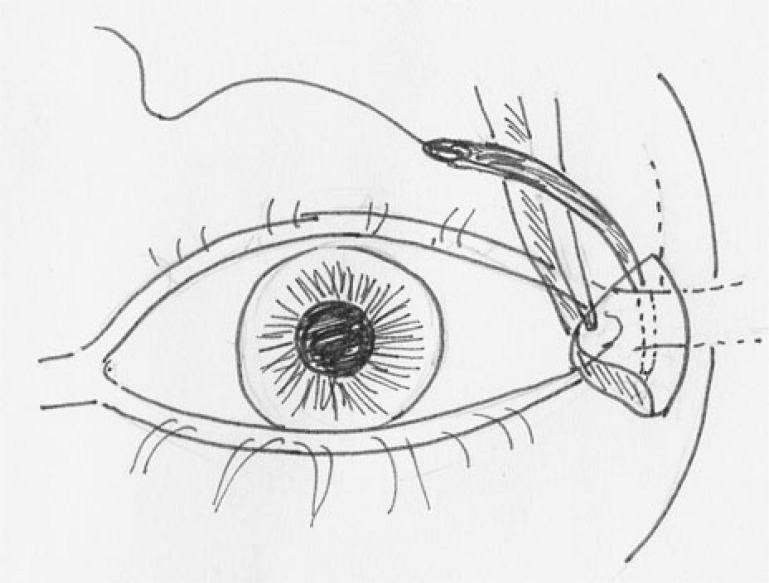
PS: The straight needle was replaced with a semicircular one and the 5:0 polypropylene suture was tied to the attachment of posterior limb of medial canthal tendon and adjoining periosteum at posterior lachrymal crest.

**Fig. (3a):**
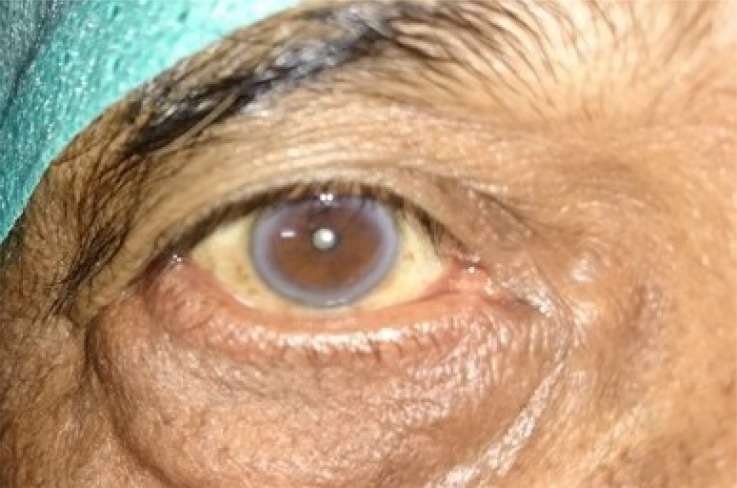
Case 1of group B: Showing preoperative lid laxity in a patient with involutional ectropion.

**Fig. (3b):**
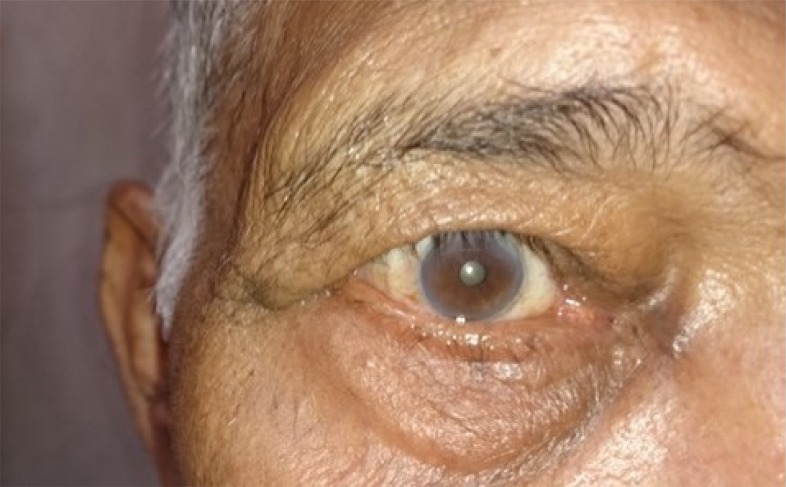
Case 1 of group B: Post- operative appearance at 1 month showing well apposed puncta.

**Fig. (3c):**
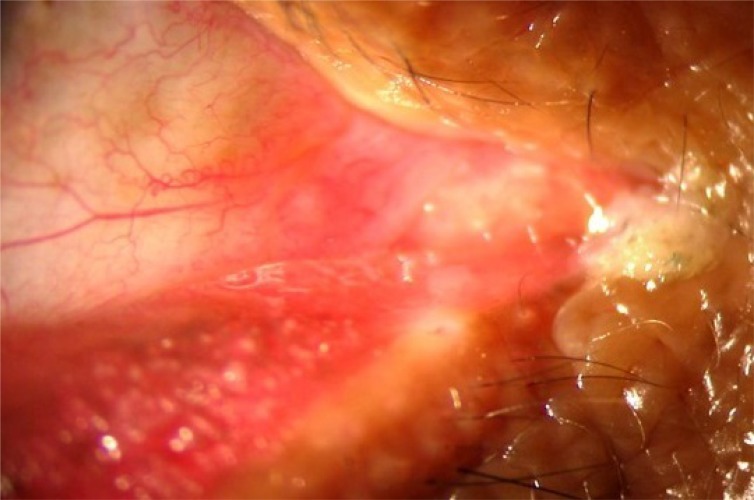
Case 1 group A: Clinical photograph showing exposed puncta preoperatively.

**Fig. (3d):**
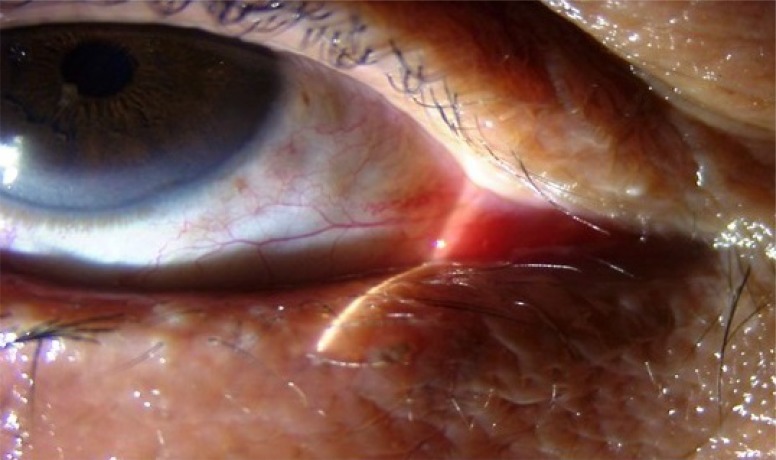
Case 1 group A: Clinical photograph at post- operative 6 month showing well apposed puncta.

**Fig. (4a):**
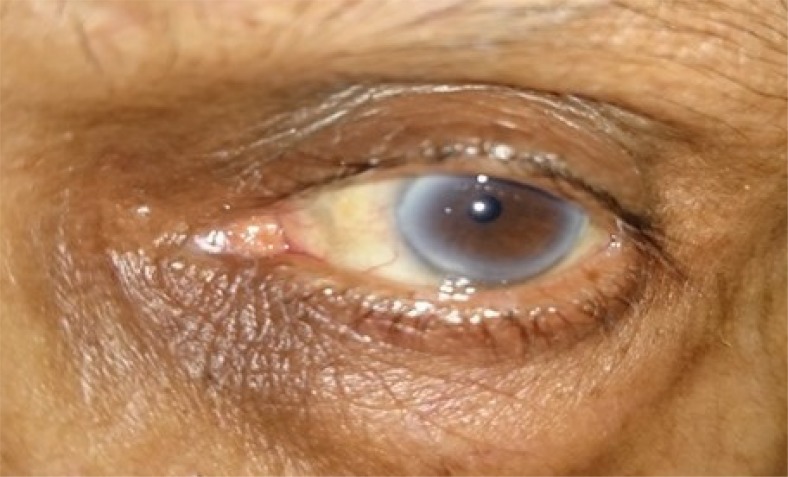
Case 2 group B: Preoperative photograph of a patient with involutional ectropion.

**Fig. (4b):**
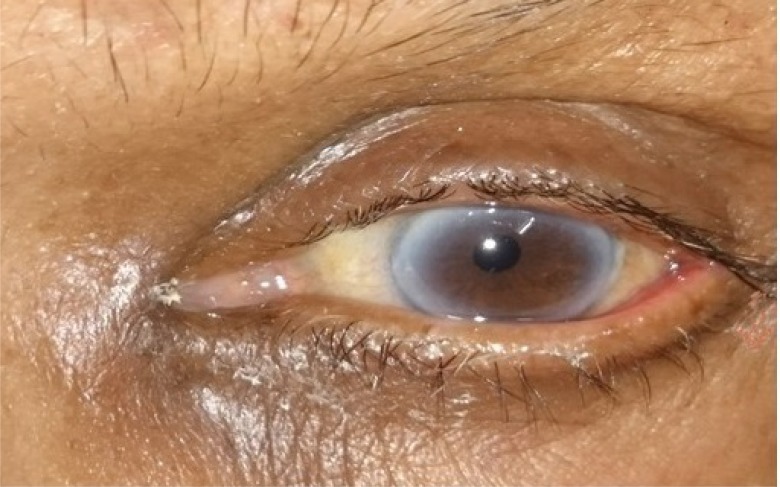
Case 2 group B: Postoperatively lateral ectropion developed in the patient noticed on postop day1.

**Fig. (5a):**
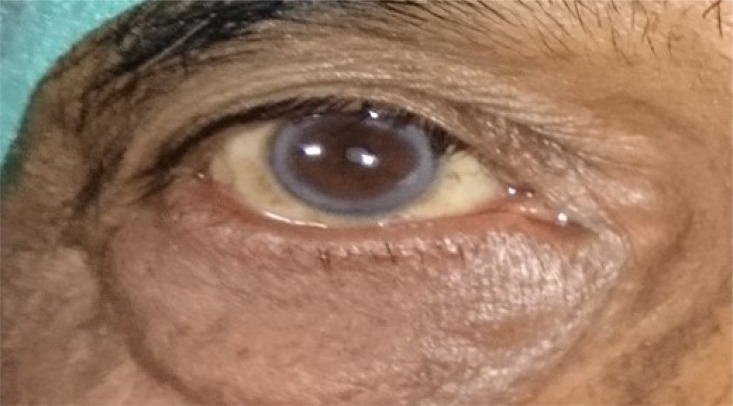
Case 3 group B: Preoperative lid laxity in a patient with involutional ectropion.

**Fig. (5b):**
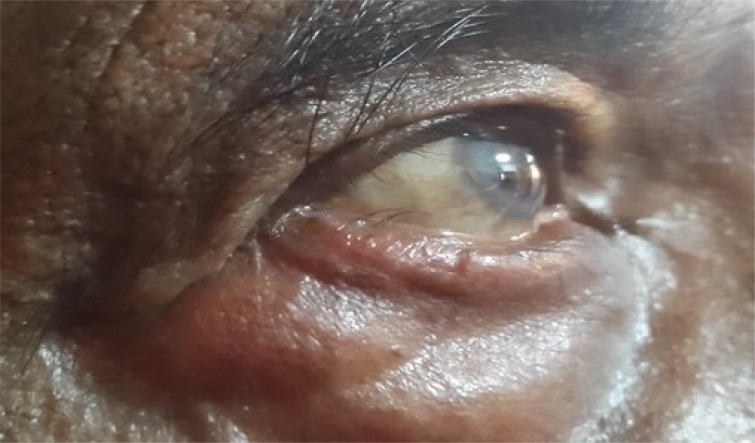
Case 3 group B: Postoperative lateral entropion in the same patient that developed at 5th month of follow up.

**Table 1:** The number of patients with different grades of ectropion in the two groups.

**Grade**	**Group A**		**Group B**		**p-Value**	**Total**
	**n**	**%**	**n**	**%**		
1	3	20.00%	6	40.00%	0.228	9.000
2	4	26.67%	0	0.00%	0.116	4.000
3	2	13.33%	0	0.00%	0.044	2.000
4	5	33.33%	8	53.33%	0.028	13.000
5	1	6.67%	1	6.67%	0.008	2.000
**TOTAL**	**15**	**100%**	**15**	**100%**		
Mean	2.80		2.87			
±sd	1.32		1.60			
p-value	0.451					

**Table 2:** Final outcome in the 2 groups at 12 months. (A: apposed; Na: not apposed).

	PUNCTA		HORIZONTAL LAXITY(mm)		RESULT
	**Preop**	**12^th^ month**	**Preop**	**12^th^ month**	
GROUP-A LTS WITH TRP	A	A	10	2	SUCCESSFUL
	Na	A	10	4	SUCCESSFUL
	A	A	11	3	SUCCESSFUL
	A	A	13	4	SUCCESSFUL
	Na	A	12	4	SUCCESSFUL
	Na	A	12	2	SUCCESSFUL
	Na	A	12	2	SUCCESSFUL
	Na	A	12	2	SUCCESSFUL
	A	A	13	3	SUCCESSFUL
	A	A	10	6	FAILED
	A	A	10	3	SUCCESSFUL
	A	A	12	4	SUCCESSFUL
	A	A	12	2	SUCCESSFUL
	A	A	10	2	SUCCESSFUL
	A	A	10	4	SUCCESSFUL
	A	A	10	4	SUCCESSFUL
	Na	A	13	2	SUCCESSFUL
	Na	A	11	4	SUCCESSFUL
	Na	A	12	4	SUCCESSFUL
	A	A	10	10	FAILED
	A	A	10	2	SUCCESSFUL
GROUP B PS	A	A	10	4	SUCCESSFUL
	Na	A	11	8	FAILED
	A	A	10	2	SUCCESSFUL
	Na	A	11	2	SUCCESSFUL
	Na	A	11	4	SUCCESSFUL
	Na	A	11	2	SUCCESSFUL
	Na	A	11	2	SUCCESSFUL
	Na	A	11	4	SUCCESSFUL
	A	A	9	2	SUCCESSFUL
